# A Whodunnit: Anterior ST-Elevation Myocardial Infarction in a Patient Found to Have Apparent Takotsubo Cardiomyopathy, Left Anterior Descending Artery Disease, and Myocardial Bridging

**DOI:** 10.7759/cureus.75037

**Published:** 2024-12-03

**Authors:** Justin Salman, Ariella Azimi, Shehab Al Ansari, Kevin Honan, Salman A Arain

**Affiliations:** 1 Department of Internal Medicine, University of Texas Health Science Center at Houston, Houston, USA; 2 Department of Internal Medicine, Methodist Health System, Dallas, USA; 3 Department of Cardiovascular Medicine, University of Texas Health Science Center at Houston, Houston, USA

**Keywords:** cardiac chest pain, intramyocardial bridging, myocardial ischemia, st-elevation myocardial infarction (stemi), st elevations, takotsubo cardiomyopathy

## Abstract

We present a case of a 52-year-old male with no known past medical history who presented to an outside hospital with acute chest pain. Initial workup revealed anteroseptal ST-elevation myocardial infarction (STEMI) for which the patient was transferred to our facility for emergent percutaneous coronary intervention (PCI). However, the patient’s hospital course revealed numerous confounding pathologies that can also present as STEMI, including transthoracic echocardiogram (TTE) abnormalities consistent with takotsubo cardiomyopathy (TCM) as well as myocardial bridging presenting as post-PCI STEMI in the setting of nitroglycerin use.

The purpose of this case report is to discuss non-acute coronary syndrome causes of STEMI, specifically TCM and myocardial bridging, in a patient who presented with acute onset chest pain and ECG changes.

## Introduction

While acute coronary syndrome (ACS) is the primary concern for acute ST elevation changes on ECG, non-ACS ST-elevation myocardial infarction (STEMI) mimics must be considered [1,2]. These include pericarditis, myocarditis, Brugada syndrome, coronary vasospasm, and coronary artery aneurysm/dissection [2]. Prompt recognition of these conditions is vital for management, although diagnosis in an emergent setting can be challenging. Takotsubo cardiomyopathy (TCM), or stress cardiomyopathy, and myocardial bridging are two such pathologies that can present similarly to STEMI. TCM involves acute transient left ventricular (LV) systolic dysfunction thought to be neuro-hormonally mediated and triggered by a stressful event; it can mimic STEMI both in clinical presentation and on ECG [3,4]. Similarly, myocardial bridging is a congenital anatomic defect where the left anterior descending (LAD) artery is covered by myocardial tissue [5]. In certain conditions, such as the administration of nitroglycerin, it can resemble a STEMI by augmenting myocardial contractility and exaggerating the systolic compression of the coronary artery causing vessel occlusion [5,6]. We present a case of a 52-year-old male who presented with chest pain with findings concerning for myocardial infarction, TCM, and myocardial bridging.

## Case presentation

**Figure 1:**
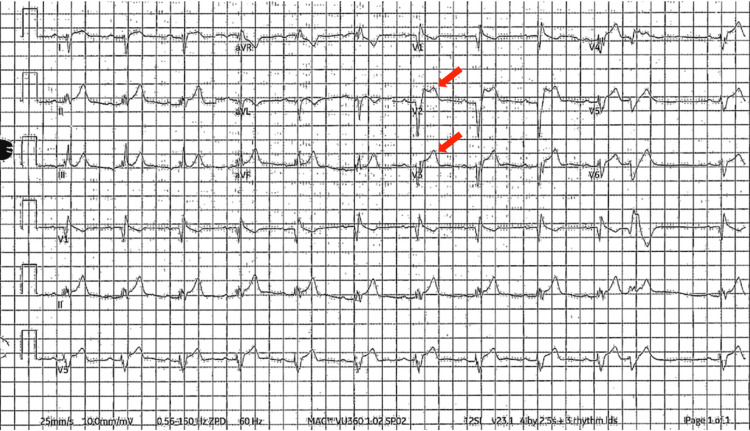
ECG showing ST elevation in leads V2 and V3 (red arrows).

A 52-year-old Hispanic male with no known past medical history presented to the emergency department (ED) for acute onset chest pain that woke him up from sleep in the early morning. He described the chest pain as a constant, non-exertional, substernal pressure with radiation down both arms. The patient reported experiencing a similar episode of chest pain two weeks ago that resolved within 30 minutes after taking an aspirin and a cold shower. The patient decided to go to the ED after this most recent episode did not resolve despite attempting similar interventions. In the ED at the outside hospital, the patient was reported to have an anterior STEMI. He was given a loading dose of aspirin and clopidogrel as well as tenecteplase before being transferred via helicopter to our facility for cardiac catheterization. Initial vitals on arrival showed blood pressure of 150/106 mmHg, heart rate of 72 beats per minute, temperature of 97°F, respiratory rate of 14 breaths per minute, and oxygen saturation of 93% on room air. The physical exam was within normal limits and the initial ECG showed ST elevations in leads V2-V3 consistent with anteroseptal STEMI (Figure 1).

**Figure 2:**
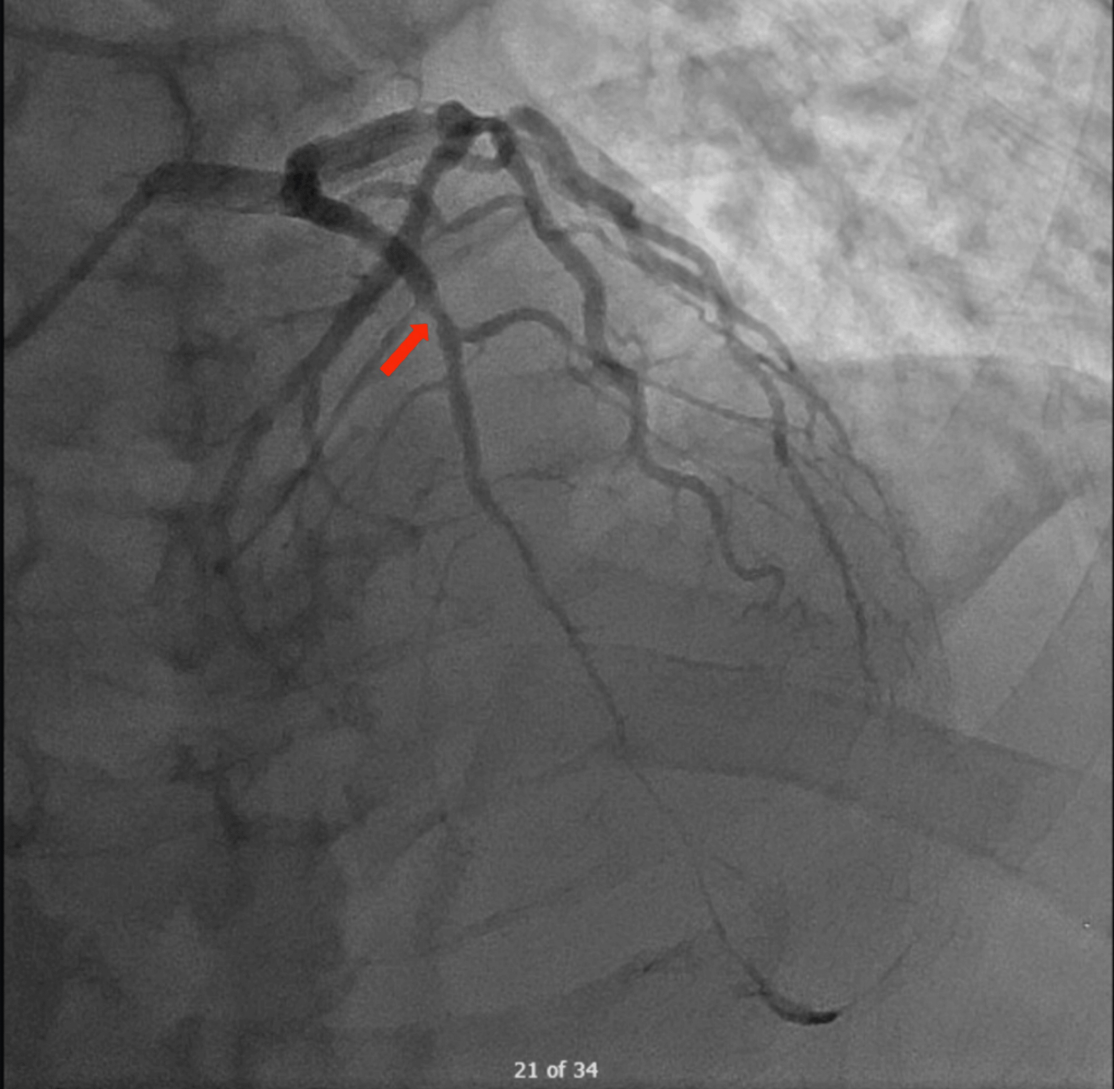
Coronary angiography of proximal left anterior descending artery lesion (red arrow).

**Figure 3:**
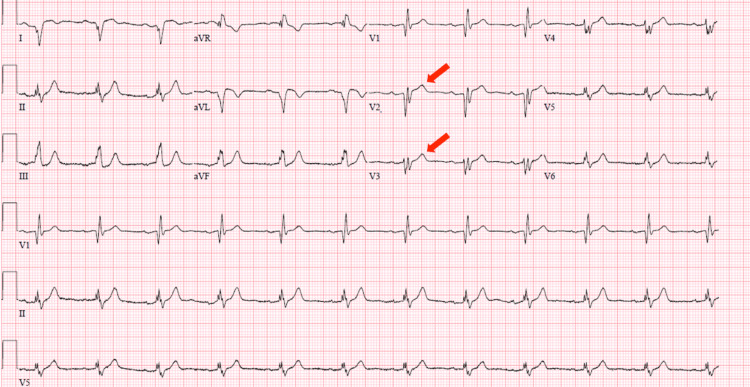
Post-percutaneous coronary intervention ECG with resolution of ST elevation in leads V2 and V3 (red arrows).

The patient was immediately taken to the cardiac cath lab where he was found to have a 70% lesion of the proximal LAD artery (believed to be the culprit lesion) with diffuse distal disease in addition to mild disease in the right coronary artery (RCA) and left circumflex artery (LCx) (Figure 2). Post-percutaneous coronary intervention (PCI) ECG showed resolution of ST elevations seen in V2 and V3 (Figure 3) and the patient was free from chest pain.

Routine labs were obtained prior to catheterization and were significant for an elevated high-sensitivity troponin I of >125,000 pg/mL (normal <= 78 pg/mL) and an elevated creatine kinase-myocardial band (CK-MB) of 828.0 ng/mL (reference range: 0.5-3.6 ng/mL).

**Figure 4:**
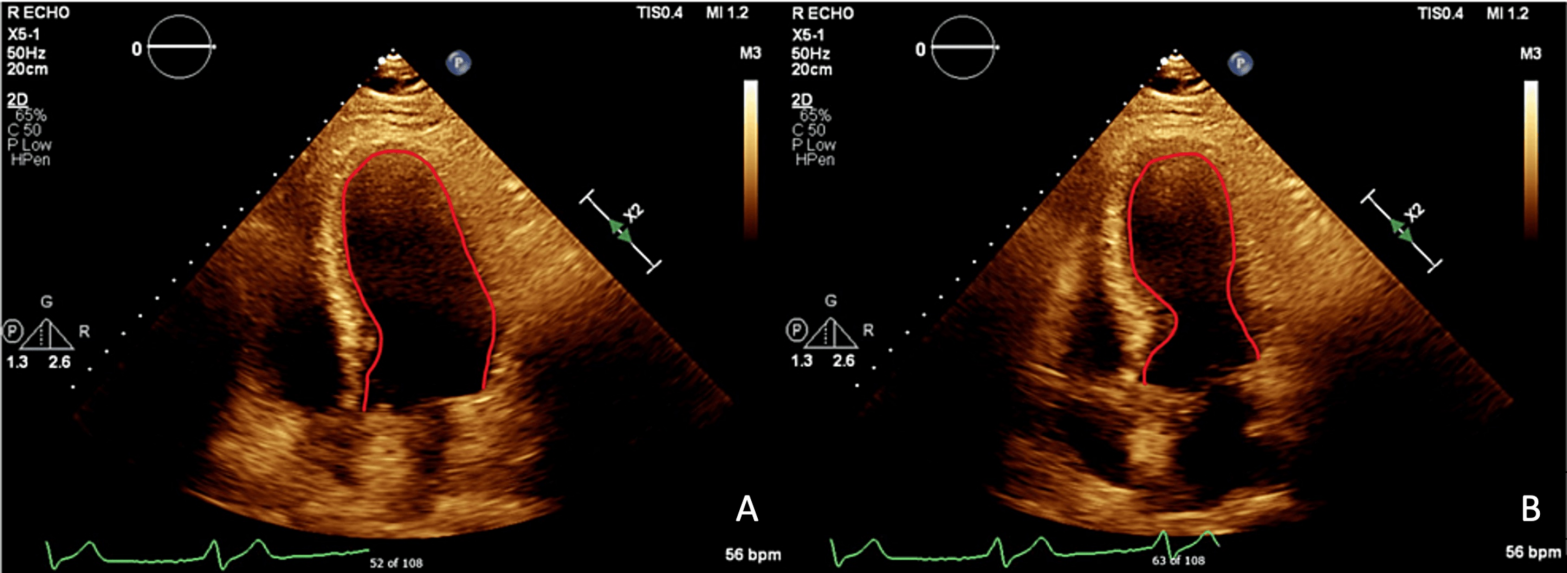
Apical four-chamber view on transthoracic echocardiogram with diastolic phase on the left (A) and systolic phase on the right (B). The red outline shows classic apical akinesis with basilar sparing and left ventricular dilatation.

The patient underwent post-PCI transthoracic echocardiogram (TTE), which demonstrated a reduced left ventricular ejection fraction (LVEF) of 30-35% with wall motion abnormalities (WMA) more consistent with TCM (Figure 4) as well as LV thrombus.

**Figure 5:**
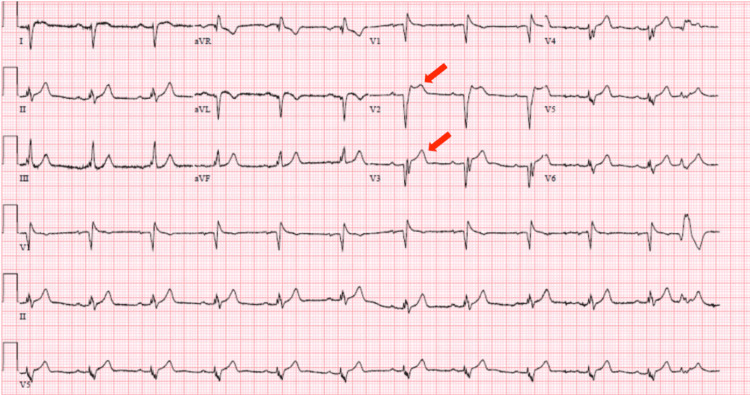
ECG showing recurrent ST elevation in leads V2 and V3 associated with chest pain after administration of nitroglycerin.

Shortly after his PCI, the patient endorsed recurrent substernal chest pain. His vitals were notable for a systolic blood pressure of 190 mmHg, therefore the patient was started on a nitroglycerin drip. Following the initiation of nitroglycerin, the patient reported worsening chest pain with evidence of a recurrent ST elevation in leads V2 and V3 on repeat ECG (Figure 5).

**Figure 6:**
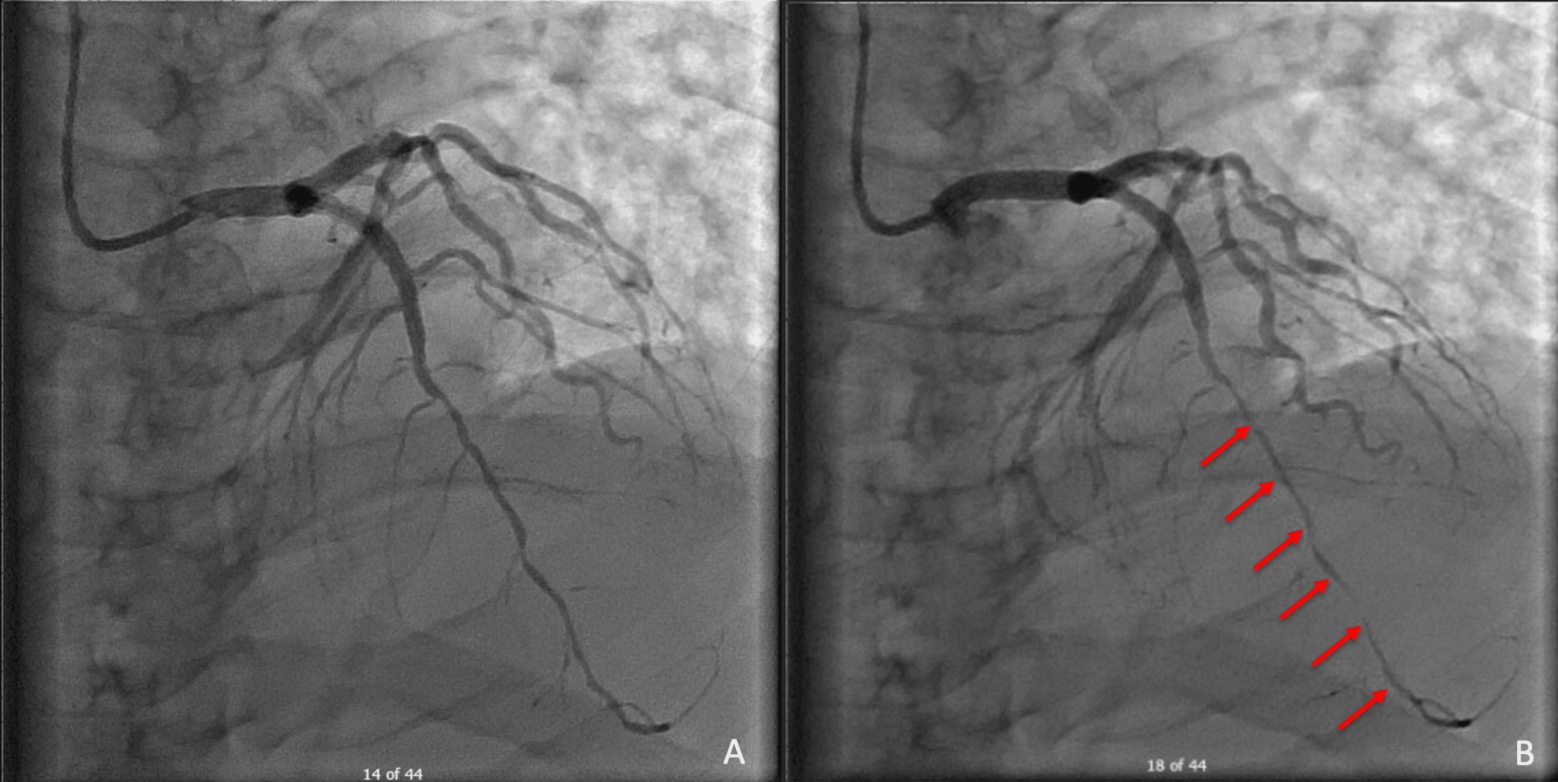
Coronary angiography showing a diastolic phase on the left (A) and a systolic phase on the right (B) with decreased vessel caliber in the mid to distal left anterior descending artery (red arrows).

The patient was emergently taken to the cath lab where he was found to have significant narrowing of the mid-distal LAD vessel caliber in systole consistent with myocardial bridging (Figure 6). Videos 1, 2 show a live-action representation of the patient's initial left heart catheterization (LHC) before and after PCI. Video 3 shows the subsequent LHC after the patient had recurrent chest pain and EKG changes, which shows mid-LAD segment bridging with vessel narrowing in systole.

**Video 1:** Initial left heart catheterization before percutaneous coronary intervention.

**Video 2:** Initial left heart catheterization after percutaneous coronary intervention.

**Video 3:** Repeat left heart catheterization showing myocardial bridging of the mid-left anterior descending (LAD) artery.

**Figure 7:**
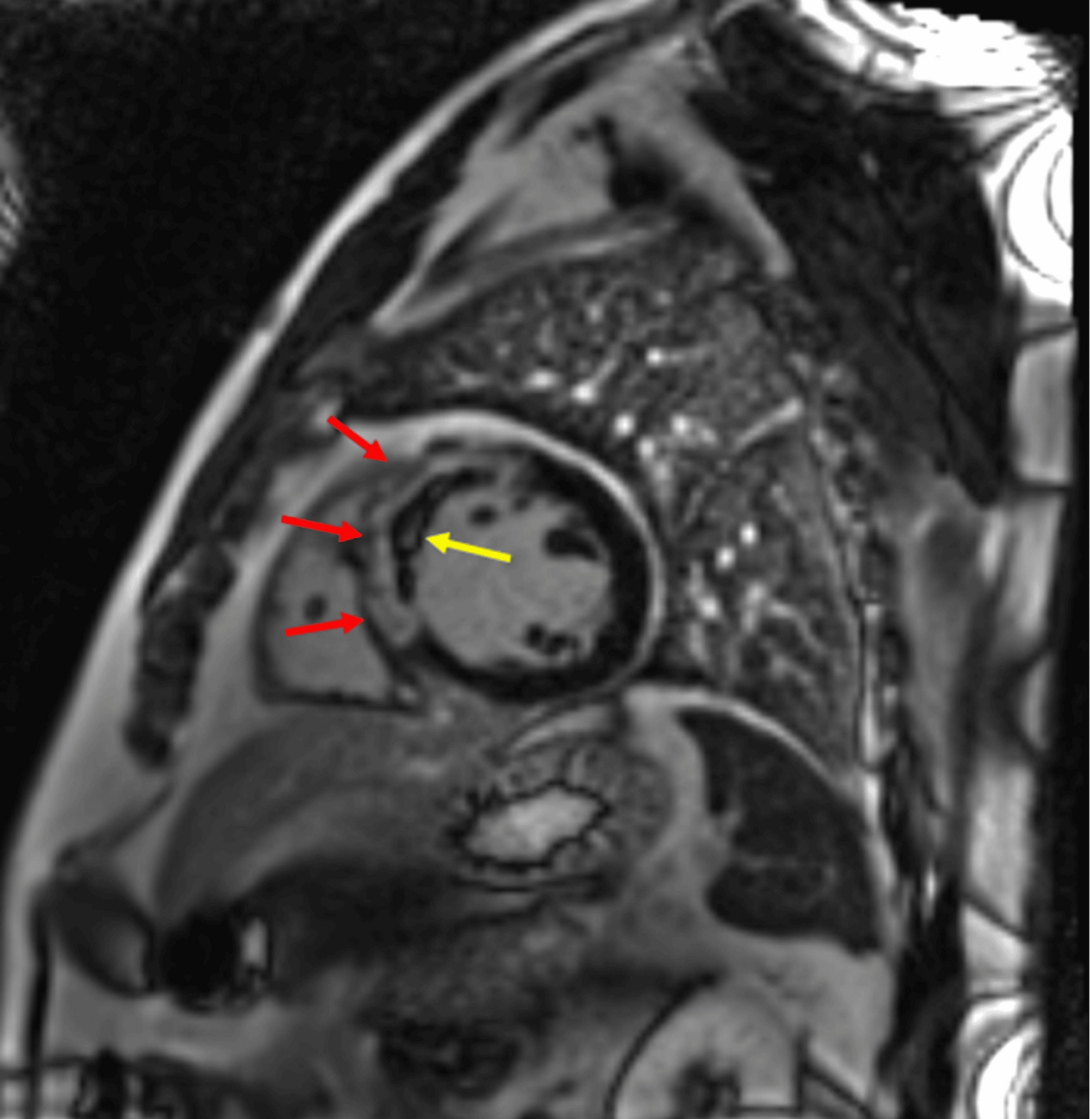
Sagittal view of cardiac MRI on the left showing late gadolinium enhancement (red arrows) with black core (yellow arrow) in the anteroseptal wall of the left ventricle suggestive of myocardial ischemia with microvascular occlusion.

Nitroglycerin was stopped as it was believed to be the offending agent, and the patient was placed on an intra-aortic balloon pump due to concern for cardiogenic shock post STEMI but was quickly weaned off the following day. He underwent cardiac magnetic resonance imaging (MRI) to further characterize a suspected LV thrombus seen on the initial TTE. Cardiac MRI demonstrated transmural late gadolinium enhancement (LGE) at the mid-apical septum as well as the anterior and apical left ventricle without evidence of LV thrombus (Figure 7).

The patient was discharged on hospital day five and was seen in the clinic one month after discharge with the resolution of his chest pain. Follow-up TTE six months post PCI showed persistent heart failure with reduced ejection fraction (HFrEF) of 35-40% with LV apical and anterior WMA.

## Discussion

STEMI on ECG is most associated with ACS events. However, there are multiple STEMI mimics that also require appropriate consideration. In this case, the patient was found to have significant LAD disease, but his hospital course revealed potential alternative causes for his clinical presentation with the discovery of myocardial bridging, which was discovered when the patient developed a STEMI after the administration of nitroglycerin. Myocardial bridging, once thought to be a benign anatomic variant, has an incidence rate of 16-40% [7]. ACS presentation in patients with myocardial bridging who receive nitroglycerin is thought to be due to a combination of augmented systolic compression causing exaggerated vessel collapse and coronary vasospasm during diastole when coronary perfusion occurs [6,8]. Additionally, studies have shown that myocardial bridging can cause accelerated development of atherosclerotic plaques proximal to the bridged areas through abnormal wall stress leading to endothelial dysfunction [9,10]. Conversely, bridging exhibits a protective effect on tunneled segments, though the exact mechanism is not well elucidated [11]. To further complicate this patient's clinical presentation, his initial TTE post PCI showed wall motion abnormality in a distribution consistent with TCM, which typically involves more than one epicardial vascular territory and has a characteristic apical ballooning of the left ventricle [12]. It should be noted that significant coronary artery disease does not preclude the diagnosis of TCM [3]. In this case, cardiac MRI showed a pattern of LGE representing a transmural scar of the LV in the LAD territory consistent with ST-elevation ACS [13]. TCM, conversely, appears as diffuse, transmural edema with high T2 signal intensity and does not correspond to any single coronary artery distribution [14]. Additionally LGE, once thought to be characteristically absent in TCM, can be seen on MRI although with a more variable pattern of distribution [15]. This further highlights the difficulty in distinguishing TCM from other cardiomyopathies.

## Conclusions

While ACS is the most urgent consideration for STEMI, there are non-ACS STEMI mimics that must be considered such as TCM and myocardial bridging. These diagnoses are often difficult to make and can confound initial presenting signs. Moreover, the management of each of these pathologies can often exacerbate the other, notably in the case of myocardial bridging, which can cause ischemia with the administration of nitroglycerin, a common therapy in ACS management. Therefore, it is important to consider these differentials should a patient experience persistent chest pain or if sonographic findings are incongruent with the vascular territory of a presumed culprit coronary artery.
